# Wear characteristics of an unconstrained lumbar total disc replacement under a range of *in vitro* test conditions

**DOI:** 10.1002/jbm.b.33456

**Published:** 2015-09-28

**Authors:** Philip J. Hyde, John Fisher, Richard M. Hall

**Affiliations:** ^1^ Institute of Medical and Biological EngineeringUniversity of LeedsLeedsUK

**Keywords:** tribology, biotribology, wear, TDR, total disc replacement, Charité, Prodisc, in motion artificial disc

## Abstract

The effect of kinematics, loading and centre of rotation on the wear of an unconstrained total disc replacement have been investigated using the ISO 18192‐1 standard test as a baseline. Mean volumetric wear rate and surface morphological effects were reported. Changing the phasing of the flexions to create a low (but finite) amount of crossing path motion at the bearing surfaces resulted in a significant fall in wear volume. However, the rate of wear was still much larger than previously reported values under zero cross shear conditions. Reducing the load did not result in a significant change in wear rate. Moving the centre of rotation of the disc inferiorly did significantly increase wear rate. A phenomenon of debris re‐attachment on the UHMWPE surface was observed and hypothesised to be due to a relatively harsh tribological operating regime in which lubricant replenishment and particle migration out of the bearing contact zone were limited. © 2015 Wiley Periodicals, Inc. J Biomed Mater Res Part B: Appl Biomater, 105B: 46–52, 2017.

## INTRODUCTION

Articulating total disc replacement (TDR) for the natural intervertebral disc (IVD) was first introduced in significant numbers in the 1980s with the Link SB Charité disc (Waldemar Link Gmbh and Co., Hamburg, Germany), originally developed at the Charité Hospital, Berlin.^1^ A relatively recent version of this design (Charité, DePuy Spine, Raynham, MA) is the subject of this investigation (referred to as “Charité” throughout this article). It is an unconstrained design, meaning that the limits of motion are not mechanically limited and a certain amount of displacement is permitted in the lateral plane, facilitated by the use of a mobile central core of ultra high molecular weight polyethylene (UHMWPE). The Charité disc has now been replaced by the “In Motion” artificial disc, but the design has essentially remained the same while incorporating minor modifications to the end plates to aid insertion using instrumentation.

The bio‐tribology of replacement joints has been reported extensively and over several decades in the hip^2^, ^3^, ^4^ and knee,^5^, ^6^, ^7^, ^8^, ^9^, ^10^ but there have been fewer studies of polyethylene wear in TDR.^11^, ^12^, ^13^, ^14^, ^15^, ^16^ The different biomechanical environment and designs utilised in the spine in comparison to the hip and knee may influence wear of the polyethylene, and potential for osteolysis.^17^

The initial clinical opinion regarding articulating replacement discs was that wear of the bearing surfaces would not be a major cause of concern, because of much reduced kinematics of operation in everyday activity.^18^ However, several recent clinical research papers have highlighted adverse tissue reactions found in samples taken from failed artificial TDR procedures.^19^, ^20^, ^21^, ^22^, ^23^, ^24^, ^25^, ^26^ It has been shown that TDRs may potentially suffer from a similar failure mechanisms to other polyethylene based joint replacements in the medium to long‐term.^27^ The wear of Charité bearings simulated *in vitro* has been shown to vary from 0.12 mm^3^ wear per million cycles under zero cross shear motion path kinematics^11^ (such as those stipulated by the ASTM guidance document F2423‐05) to 20.76 mm^3^ per million cycles^16^ when anterior‐poster shear is added to the standard ISO 18192–1 test cycle. There is no data on the wear of UHMWPE TDRs under very small, but non‐zero, crossing path motions that could occur under the wide range of patient‐specific biomechanical conditions. Because of the wide variation in patient weight and muscles forces acting across the functional spinal unit, the axial loading on TDRs will probably vary accordingly between subjects. Previously, Charite TDRs have always been positioned in their test chambers with the CoR of the testing machine set to coincide with the centre of the TDR at the centre of the polyethylene core (Christian Kaddick, personal communication, May 22, 2012; Andrew Dooris, personal communication, January 22, 2014). Since the CoR of the lumbar spinal unit is usually thought to be below the centre of the natural disc,^28^ it may be more appropriate to position the device *in vitro* so as to investigate the influence of a more physiological CoR.

The aim of the study presented here was to compare the wear characteristics of Charité TDRs under a range of kinetic conditions, starting with the parameters used in the ISO 18192‐1 standard as the baseline study. Specific research questions arising from the literature were:

1. What is the effect of reduced (rather than zero) cross shear kinematics at the bearing surface when the ISO 18192‐1 standard motions are used subject to a changed phasing of the flexion and lateral bend motions?
2. What influence does a reduction in loading cycle have on wear rate when the other ISO standard inputs remain unchanged?
3. What effect does changing the position of the CoR from a central position (previous tests) to one inferior to the device (i.e., physiologically relevant) have on the wear rate?

## METHODOLOGY

**Figure 1:**
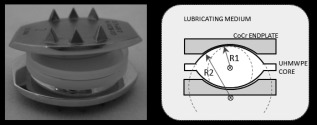
Charité TDR showing CoCrMo endplates sandwiching the mobile UHMWPE core (left) and schematic (right) showing the position of the CoR used for the wear test studies.

Four individual wear test regimes were applied to Charité TDR components: standard ISO 18192‐1, low cross shear (LXS), lowered axial loading (LL) and changed CoR position (ΔCoR). A six‐station Leeds SimSol spine simulator (Simulation Solutions Ltd, Stockport, UK) was used throughout. A detailed methodology has been described in detail elsewhere.^14^ A total of 10 commercially available Charité (DePuy Spine, Reynham, MA) lumbar disc (Figure 1, left) were used for wear testing. In the first four experiments six TDRs were tested kinematically and one was used as a dynamically loaded soak control. For the final ISO test (ISO3) and following **“**changed CoR” study (ΔCoR) fresh discs were used (*n* = 3). The samples used were **“**size 2” with core heights of 7.5 mm and radius 13 mm with matching cobalt chromium molybdenum (CoCrMo) endplates. The manufacturer states that the Charité UHMWPE bearing core is manufactured from GUR1020 and is gamma sterilised at 2.5–4 MRads.

The kinematic and load cycles used in this study were founded on the ISO standard 18192‐1 (referred to as ISO, ISO2 or ISO3 which were all identical tests) and considered to be the baseline test (Table 1). Subsequent alterations to these baseline parameters used to investigate effect on the rates of wear are listed in Table 1. At each 1 million cycle point the simulator test cells were completely stripped and cleaned utilising an enhanced protocol.^14^ The Charité components were then stored in a temperature controlled measurements lab for stabilisation for 48 h. At this point gravimetric measurements were completed (Mettler AT21 balance Leicester, UK, 0.001 mg resolution).

**Table 1:** Experimental Inputs Used for a Range of Testing Conditions on the Unconstrained Total Disc Replacement (parametric changes are highlighted)

Input	Study	Length (Millions Cycles)	Sample Number (*n*)	Input Parameter	Input Magnitude	Freq (Hz)	Phase wrt FE (°)	CoR POSITION (wrt Figure 1)
ISO, ISO2, ISO3	Baseline ISO input and repeats	4	6	AF	600–2000 N	2	N/A	R1
		2	6	AR	+2°/−2°	1	+90	
		2	3	FE	+6°/−3°	1	0	
				LB	+2°/−2°	1	−90	
LXS	Low cross shear	2	6	AF	600–2000 N	2	N/A	R1
				AR	+2°/−2°	1	+90	
				FE	+6°/−3°	1	0	
				LB	+2°/−2°	1	0	
LL	Low load	4	6	AF	300–1000 N	2	N/A	R1
				AR	+2°/−2°	1	+90	
				FE	+6°/−3°	1	0	
				LB	+2°/−2°	1	−90	
ΔCoR	Changed centre of rotation position	2	3	AF	600–2000 N	2	N/A	R2
				AR	+2°/−2°	1	+90	
				FE	+6°/−3°	1	0	
				LB	+2°/−2°	1	−90	

The low cross shear (LXS) experiment was designed to test the effect on wear when there was a small, but finite, amount of crossing path motion at the bearing surface. To do this the FE and LB motions were changed from 90° out of phase to 0° in phase while leaving the axial rotation (AR) input 90° out of phase with the other two articulations. This changed the open elliptical motion path to a narrow elliptical path. Following this the ISO standard was again used but this time with the AF input load reduced by 50%. In the final study using *n* = 6 the ISO test was repeated (ISO2) to check repeatability of the simulator. For the final experiment three new discs were used and the ISO standard again used as a baseline test (ISO3). Following this the CoR position was changed to be inferior to the lower endplate (R2 in Figure 1), as is the case *in vivo*.^29^ The radius (R2) of the Charité endplate bearing face was 13 mm, thus the CoR was chosen to be 13 mm below the upper endplate bearing rather than 3.75 mm as has been used previously in the literature (R1 in Figure 1).

**Figure 2:**
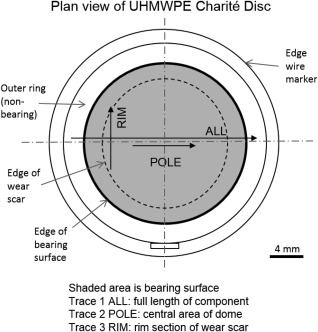
Surface profilometry trace positions over the UHMWPE core component.

A contacting profilometer (Form Talysurf series, Taylor Hobson, UK) was used to record changes in surface topography after each study. Areas of interest on the UHMWPE core were differentiated according to Figure 2. The CoCrMo endplate bearing surfaces were assessed using a single full trace from edge to edge. Filtering was by Gaussian filter with cut‐off values of 0.8 and 0.25 mm for the UHMWPE core and CoCrMo bearings respectively at a ratio 100:1. On the ISO, LXS, LL, and ISO2 tests statistical analysis used one way ANOVA with *post hoc* tests using repeated measures (Bonferroni, *α* = 0.05). A paired *t* test was used for the ISO3 and ΔCoR study.

Optical microscopy was completed using a stereo microscope (Leitz Laborlux 12 ME ST) at 25× magnification and images recorded using a digital camera (Olympus Camedia C‐5050). Scale bars on the images were calibrated using a 1 mm graticule. SEM images were taken using a Phillips XL30 SEM used in secondary electron mode at 20 KV acceleration. UHMWPE cores were gold coated (∼20 nm) and mounted on aluminium stubs with carbon paint to help ensure efficient discharging at the surface. Magnifications between 20× and 5000× were obtained.

## RESULTS

**Figure 3:**
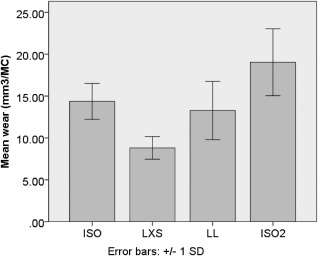
Rates of wear for a Charité TDR tested under ISO standard, low cross shear (LXS) and low load (LL) with a final repeated ISO standard test.

**Figure 4:**
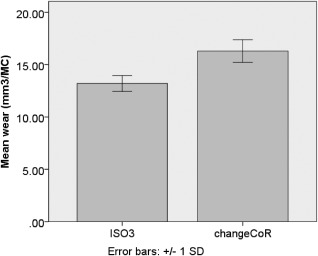
ISO standard experiment (ISO3) followed by a repeated test where the CoR of the bearing was shifted inferiorly.

The baseline rate of wear derived using standard ISO conditions was 14.4 ± 2.1 mm^3^ (± StD) per million cycles (Figure 3). Wear rate was reduced significantly (*p* < 0.01)% to 61% of the baseline result when changed to a lower cross shear (LXS) input (8.8 ± 1.4 mm^3^). Wear rate was 92% of baseline (13.3 ±3.5 mm^3^) when the AF input cycle was lowered to 50% of that indicated by the ISO standard, though this was not a significant change (*p* = 1.00). The final study on these test components was a repeatability test using the ISO standard (ISO2) which returned a similar wear rate (19.0 ± 4.0 mm^3^) to the first ISO test (*p* = 0.09), although larger variance was evident, perhaps due to the accumulated wear damage on the bearing. The following experiment utilised three new Charité samples and began with a baseline ISO standard test (ISO3). Wear rate significantly increased (*p* = 0.02) from 13.2 ± 0.8 mm^3^ to 125% of baseline (16.3 ± 1.1 mm^3^) when the CoR position was moved (ΔCoR) from the centre of the UHMWPE core to inferior to the lower baseplate (Figure 4).

**Figure 5:**
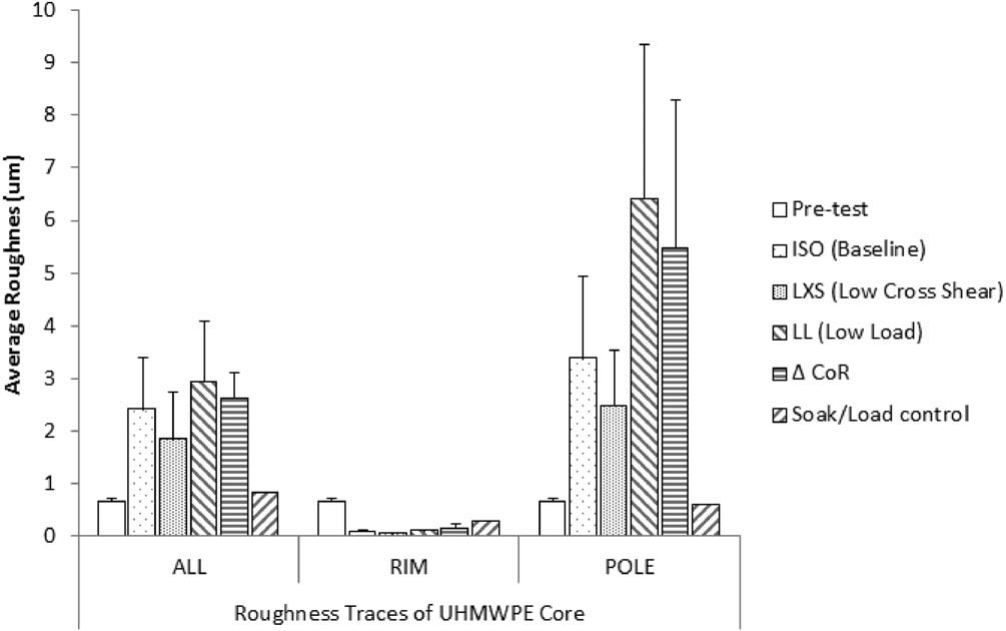
Surface topography of the UHMWPE core separated into three trace areas (according to Figure 2).

The visual appearance of the UHMWPE core of the Charité bearing changed over the course of experimentation. The unworn discs had an average roughness (Ra) value of 0.66 µm and periodic waveform which reflected the machined nature of the surface. Throughout the wear studies the average Ra value for the full disc width (ALL, Figure 2) increased significantly over the baseline test but did not change significantly between subsequent studies (Figure 5). Qualitative visual inspection indicated that the wear scars were isotropic in the radial direction from the pole to perimeter. However, the perpendicular traces (RIM, Figure 2) indicated a smoothing of the surface in that direction (RIM, Figure 5). The central areas (POLE, Figure 2) of the UHMWPE cores were generally roughened more than the surrounding surface and elevated above the mean surface form (POLE, Figure 5). Although the ISO and LXS tested discs did not show a significant change in pole area Ra compared to the rest of the disc surface, some roughening at the pole area was still observed. The single soak control disc was measured in the same way and also showed a threefold reduction in Ra at the rim areas but no roughening at the pole (Figure 5). Over 13 million cycles the average Ra of the metallic cups did not change significantly.

**Figure 6:**
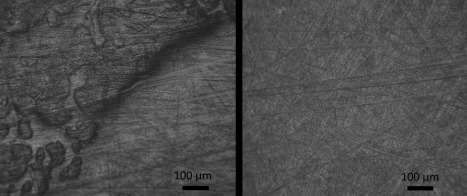
Micrograph images of a UHMWPE core after the ISO standard test (left: edge of the roughened pole area of the core; right: mid 1/3rd of the core).

High magnification micrographs showed that the surface of the UHMWPE was covered in very fine curvilinear abrasive scratches (Figure 6). Magnification of the edge of the roughened area on the pole region of the UHMWPE core (Figure 6, left), shows the apparent reattachment of wear debris.^14^

**Figure 7:**
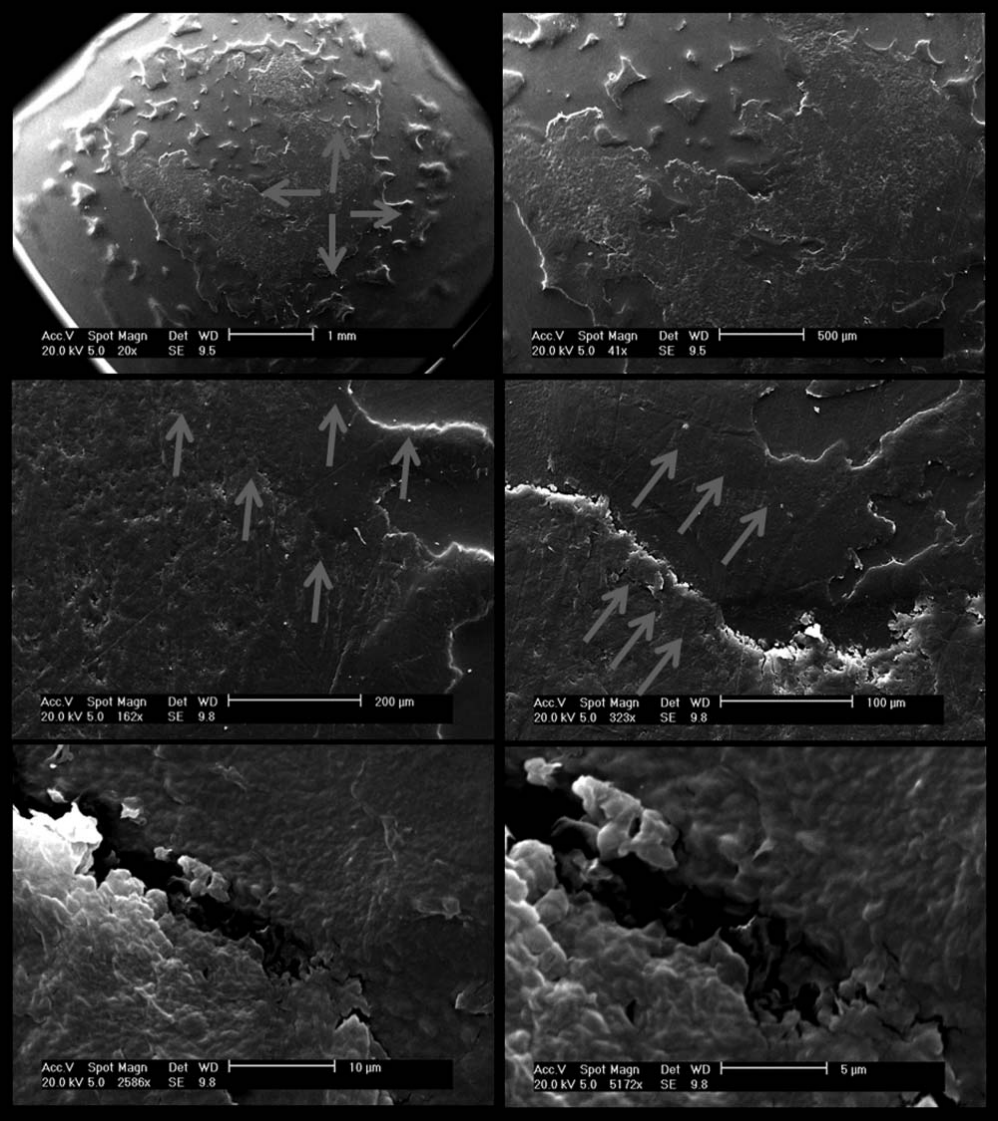
Charité SEM images (top to bottom: increase in magnification) of roughened area around the UHMWPE pole region showing appearance of built up surface layers (direction arrowed) and islands.

Secondary electron microscopy was used to further enhance the micrograph detail. The SEM images presented in Figure 7 show an increasing magnification of the debris at the pole area. There appears to be a “transfer” effect, where several layers of UHMWPE have adhered onto the surface, consisting of approximately micron‐sized particles creating layers at the centre and island features surrounding.

## DISCUSSION

Charité TDRs were subject to four wear test regimes: ISO standard 18192‐1 (ISO, ISO2, ISO3: identical baselines), low cross shear (LXS), lowered axial loading (LL) and changed CoR position (ΔCoR). The ISO standard test produced a level of wear similar to that reported in the literature.^16^ Using an input cycle with a small amount of cross shear motion (LXS) did reduce wear rate significantly, however, the amount of reduction was small compared to negligible wear reported by Serhan et al.^30^ when purely curvilinear input motions were used. Thus, wear rate of the Charité disc increases rapidly when the cross shear ratio increases from zero to a small but finite amount. A similar pattern of rapidly increasing wear with increasing cross shear ratio has been observed in UHMWPE pin‐on‐plate experiments by Kang et al.^31^ There is no previous literature describing unconstrained TDR bearing wear behaviour for kinematic inputs that lie between fully curvilinear (negligible wear) and ISO standard (highly crossing path motions and high wear). Patient disc kinematics *in vivo* are likely to be widely varied between possible extremes and this work has highlighted that even small amounts of crossing path motion will produce non‐trivial amounts of wear volume and hence particulate debris with the associated risk of osteolysis, which should be considered at the design stage of further TDR evolution.

Although Charité wear was lower when using a 50% reduced load cycle, this was not significantly different to the baseline ISO test and therefore produced relatively high wear rates. This nonlinearity between rate of wear and load applied complements the results of simple pin‐on‐plate testing configurations where polyethylene bearing contact area has been observed to be the main driver of wear factor changes rather than the load or contact pressure applied.^32^, ^33^, ^34^ It is therefore unlikely that a patient with a substantially different body weight would have an appreciably different wear rate for their TDR device.

Previous *in vitro* testing has been completed by using CoR placed at the centre of the TDR construct, that is, at the centre of the UHMWPE core.^11^, ^16^ *In vivo*, the precise CoR is difficult to determine, but in the natural intervertebral disc it is known to be inferior to the lower vertebral endplate.^35^ To test the hypothesis that CoR position may have an impact on wear behaviour of the Charité device, the CoR was changed to be inferior to the bottom endplate, at a distance of 13 mm below the upper endplate cup bearing surface. The Charité device showed a significant but modest increase in wear as a result of this change. Preclinical testing of orthopaedic implants should aim to cover an envelope of possible conditions and therefore go beyond minimum compliance to enhance safety.^36^ Considering patient spinal biomechanics vary considerably, CoR may be a pertinent testing parameter to consider when designing *in vitro* experiments for replacement discs.

The isotropic wear scars observed on the UHMWPE core were most probably because of rotation of the unconstrained core during operation^37^ which gave even wear characteristics in all directions. Lower Ra at the perimeter rim was indicative of burnishing where edge‐loading caused by the metallic endplates polished the perimeter portions of the core.^15^ There was no significant change in surface topography or appearance during the changed CoR test. Rim impingement observed in explanted devices^38^, ^39^, ^40^, ^41^ was not replicated under these conditions or when anterior shear displacement was added in a study by Vicars et al.^16^

The appearance of the SEM images (magnification 323X, Figure 7) shows similarity to an *ex vivo* UHMWPE component (Prodisc‐L) described by Choma et al.^42^ (page 293, Figure 8, right). This also displayed a similar “transfer effect,” perhaps also because of the same biomechanical reasons of small articulations and consequent debris reattachment. Conversely, an SEM image by Anderson at al.^39^ (page 111, Figure 1) of an UHMWPE explant did not indicate this phenomenon, but did have other similar features to those shown in the micrograph presented above (Figure 6, left) such as abrasive linear scratching. A similar pattern of raised roughening and “islands” of transferred debris was observed by Liao et al.^43^ when examining UHMWPE hips tested in 25% serum. The authors observed that the effect diminished when the serum concentration was 90%. The burnishing at the rim of the UHMWPE core was probably caused by an edge‐loading effect due to the adjacent CoCr bearing.^15^ Wear debris reattached to the pole area of the mobile bearing was indicative of a harsh tribological regime in which lubricant replenishment was reduced at the centre of the bearing couple lowering the rate of removal of the particulate debris into the bulk lubricating medium, in part due to smaller stroke lengths compared to similar diameter hip replacements where rotation inputs are much larger.

During this study, over a wide envelope of testing inputs, the Charité TDR rates of wear were approximately between 8 and 18 mm^3^/million cycles, which is a figure usually deemed acceptable for hip and knee replacements. However, the reaction to particulate wear debris in close proximity to the spinal canal, in a smaller joint domain, remains uncertain; reports of osteolysis have heightened the importance of this issue.^20^, ^24^, ^25^, ^26^ It remains to be seen if osteolysis will be a rare mode of failure, or, if these effects are merely in stasis at present. A recently published conference article on TDR wear debris^27^ reported a similarity to hip and knee particle morphology and is therefore a long‐term concern. Further work on biological reactivity of *in vitro* gathered lumbar TDR wear debris has been reported separately.^44^
